# Safety and efficacy of electrical stimulation for lower-extremity muscle weakness in intensive care unit 2019 Novel Coronavirus patients: A phase I double-blinded randomized controlled trial

**DOI:** 10.3389/fmed.2022.1017371

**Published:** 2022-12-06

**Authors:** Alejandro Zulbaran-Rojas, Ramkinker Mishra, Naima Rodriguez, Rasha O. Bara, Myeounggon Lee, Amir Behzad Bagheri, James P. Herlihy, Muhammad Siddique, Bijan Najafi

**Affiliations:** ^1^Interdisciplinary Consortium on Advanced Motion Performance (iCAMP), Division of Vascular Surgery and Endovascular Therapy, Michael E. DeBakey Department of Surgery, Baylor College of Medicine, Houston, TX, United States; ^2^Department of Pulmonary Critical Care, Baylor College of Medicine, Houston, TX, United States

**Keywords:** COVID-19, critically ill patients, lower extremity weakness, electrical stimulation, intensive care unit

## Abstract

**Background:**

Intensive care unit (ICU) prolonged immobilization may lead to lower-extremity muscle deconditioning among critically ill patients, particularly more accentuated in those with 2019 Novel Coronavirus (COVID-19) infection. Electrical stimulation (E-Stim) is known to improve musculoskeletal outcomes. This phase I double-blinded randomized controlled trial examined the safety and efficacy of lower-extremity E-Stim to prevent muscle deconditioning.

**Methods:**

Critically ill COVID-19 patients admitted to the ICU were randomly assigned to control (CG) or intervention (IG) groups. Both groups received daily E-Stim (1 h) for up to 14 days on both gastrocnemius muscles (GNMs). The device was functional in the IG and non-functional in the CG. Primary outcomes included ankle strength (Ankle_s_) measured by an ankle-dynamometer, and GNM endurance (GNM_e_) in response to E-Stim assessed with surface electromyography (sEMG). Outcomes were measured at baseline, 3 and 9 days.

**Results:**

Thirty-two (IG = 16, CG = 16) lower extremities in 16 patients were independently assessed. The mean time between ICU admission and E-Stim therapy delivery was 1.8 ± 1.9 days (*p* = 0.29). At 3 days, the IG showed an improvement compared to the CG with medium effect sizes for Ankle_s_ (*p* = 0.06, Cohen’s *d* = 0.77) and GNM_e_ (*p* = 0.06, *d* = 0.69). At 9 days, the IG GNM_e_ was significantly higher than the CG (*p* = 0.04, *d* = 0.97) with a 6.3% improvement from baseline (*p* = 0.029). E-Stim did not alter vital signs (i.e., heart/respiratory rate, blood saturation of oxygen), showed no adverse events (i.e., pain, skin damage, discomfort), nor interfere with ICU standard of care procedures (i.e., mechanical ventilation, prone rotation).

**Conclusion:**

This study supports the safety and efficacy of early E-Stim therapy to potentially prevent deterioration of lower-extremity muscle conditions in critically ill COVID-19 patients recently admitted to the ICU. If confirmed in a larger sample, E-Stim may be used as a practical adjunctive therapy.

**Clinical trial registration:**

[https://clinicaltrials.gov/], identifier [NCT04685213].

## Introduction

Bed rest and immobilization are time-honored treatments for managing trauma and acute or chronic illnesses. Problems arising from this treatment modality can complicate a primary disease, worsening the initial cause of admission (1). For instance, critically ill patients who require prolonged immobilization due to intensive care unit (ICU) stay often suffer from muscle weakness (2). Particularly, this condition may originate from neuro-myogenic disturbances in lower extremities (3, 4) that, when immobilized, major pathways involving inflammation, impaired oxygen delivery, and hyperglycemia arise (5, 6). These consequences are highly prevalent among hospitalized patients with 2019 Novel Coronavirus (COVID-19) in need of intensive care (7, 8). Particularly, this population receive concomitant standard therapy of paralytics and glucocorticoids that leads to inhibition of acetylcholine receptors in the neuromuscular junctions (9); ultimately, causing deleterious effects on the musculoskeletal metabolism (10).

Recent studies have explored the physiopathology of muscle wasting in critically ill COVID-19 patients (11). Cytokine storms, C-reactive protein, and pro-inflammatory molecules are thought to be part of the biological mechanism (12). These factors may induce endothelial damage and mitochondrial autophagy leading to myofibrillar breakdown (13). In the lower extremities, these consequences can contribute to muscle atrophy, weakness, functional impairment, and persistent symptoms that can last for up to 1 year following ICU discharge (14). Eventually these symptoms can increase fall risk, lack of independence, and quality of life deterioration (15, 16). Therefore, there is a need to implement a practical solution to prevent muscle deterioration of bedbound patients, particularly those with severe COVID-19 infection.

Physical therapy (PT) greatly benefits neuromuscular outcomes in patients with muscle deconditioning and weakness (17). However, reduced personnel and resources can be a limitation for ICU COVID-19 patients. Additionally, the rapid loss of muscle mass within hours after ICU admission (5) requires an immediate approach, making this condition time-dependent. One practical solution is the use of electrical stimulation (E-Stim) therapy. This modality prevents muscle deconditioning (18), improves muscle strength, and restores functionality in ICU patients (19). While it may be a suitable treatment to facilitate the rehabilitation pathways for COVID-19 patients (20), empirical evidence is needed (11). Today, this technology has been demonstrated to improve muscle strength in ICU COVID-19 patients (21). However, there is still a lack of randomized studies (22) to confirm its efficacy. Thus, it is unclear whether this adjunctive therapy prevents lower-extremity muscle deconditioning in ICU COVID-19 patients.

This study examines the potential safety and efficacy of lower-extremity E-Stim therapy to prevent lower-extremity muscle deconditioning in ICU COVID-19 patients. We hypothesized that patients receiving short-term E-Stim therapy will show significant improvement in lower-extremity outcomes [i.e., muscle endurance, ankle strength (Ankle_s_), risk of fall] compared to those who do not receive it.

## Materials and methods

### Study design and settings

Critically ill COVID-19 patients admitted to the ICU due to acute respiratory failure at Baylor St. Luke’s Medical Center (BSLMC, Houston, TX, USA) were recruited in a phase I double-blinded randomized controlled trial. Recruitment was performed from December 2020 to March 2021 by research assistants (AZ-R and NR). The protocol of the study was registered on clinicaltrials.gov, Identifier: NCT04685213. This study followed the Consolidated Standards of Reporting Trials (CONSORT) guidelines for randomized clinical trials.

### Participants

To be eligible, patients must have been admitted to the ICU due to COVID-19 infection within 3 days prior to initiating E-Stim therapy, received assisted ventilation therapy, and indicated bed rest for at least 7 days. These conditions were based on the judgment of clinical intensivist investigators (MS and JPH). Patients were excluded if they were medically paralyzed (i.e., rocuronium, cisatracurium) or under vasopressor therapy (i.e., norepinephrine, epinephrine, vasopressin) at the moment of enrollment; expected to be discharged from critical care in the next 24 h; had below the knee amputations or lower-extremity wounds; demand-type cardiac pacemaker, implanted defibrillator, or other implanted electronic devices; and any conditions that may interfere with outcomes or increase the risk of the use E-Stim based on the judgment of clinicians.

### Intervention

**FIGURE 1:**
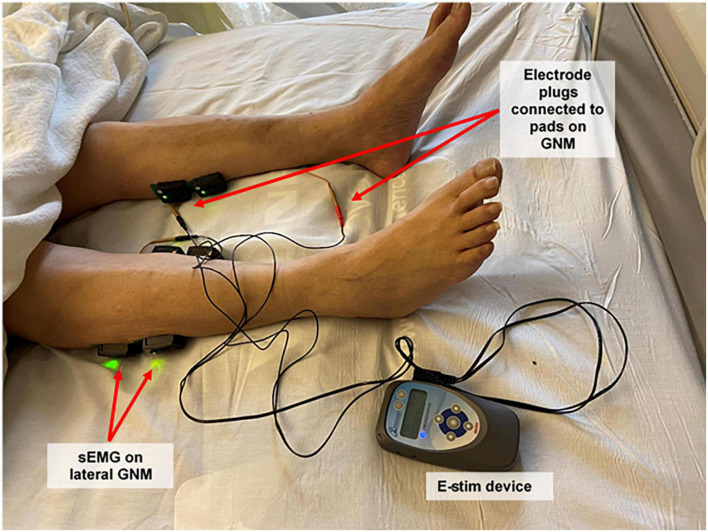
Study setup: electrical stimulation device, plugs and pads, and surface electromyography sensors. Participants received electrical stimulation through electrode adhesive pads placed on both proximal and distal gastrocnemius muscles using a bio-electric stimulation technology^®^ (BEST) micro-current platform (Tennant Biomodulator^®^). Electrical stimulation (E-Stim) was active in the intervention group and non-functional in the control group. Two surface electromyography (Delsys Trigno Wireless EMG System, MA, USA) sensors were placed on the proximal lateral gastrocnemius of each lower extremity to evaluate muscular outcomes. Proximal medial gastrocnemius signal was also recorded, but not included for analysis. sEMG, surface electromyogram; GNM, gastrocnemius muscle; E-Stim, electrical stimulation.

Patients were randomized (ratio: 1:1) to either control (CG) or intervention (IG) groups through a computer-generated list followed by sequential allocation. Participants and care providers were blinded to the group allocation. The IG received E-Stim through two electrode adhesive pads (2 cm × 2 cm, Conductive electrode pads, Avazzia Inc., Dallas, TX, USA) placed on proximal gastrocnemius muscle (GNM) (23) and Achilles tendon of each leg using a bio-electric stimulation technology (BEST^®^, Dallas, TX, USA) microcurrent platform [Tennant Biomodulator device (R), Dallas, TX, USA, Figure 1] for 1 h daily for up to 14 days. The CG was provided with an identical but non-functional device (placebo) for the same period. Therapy was delivered in supine position placing the head of the patient’s ICU bed between 30–45 degrees. In cases of prone positioning, E-Stim therapy was delivered placing the head of the patient’s ICU bed within 20° (24).

The E-Stim application was set at 50 V with an interactive high voltage pulsed alternative current (HVPAC) in the shape of an asymmetrical damped sinusoidal biphasic pulsed waveform (25), which allows for muscle relaxation and avoids fatigue during therapy (26). An intensity level from 50 to 250 V has been previously FDA-cleared for the use of pain relief (25). The pulse duration was between 400 and 1400 microseconds (μs), and pulse frequency between 20 and 121 hertz (Hz). These same intensity level and pulse characteristics were shown to be harmless in a previously published clinical trial for lower-extremity ischemic lesions (27). E-Stim was discontinued if the patient presented rapid deterioration [i.e., arterial blood oxygen desaturation < 93% under ventilation assistance, hemodynamic instability, septic shock, thigh extracorporeal membrane oxygenation (ECMO) placement, or generalized gross edema] despite intensive care treatment. Intubation was not an indication for E-Stim discontinuation.

### Equipment for muscular assessment and data analysis

Surface Electromyography (sEMG, Delsys Trigno Wireless EMG System, MA, USA) was recorded bilaterally from the proximal lateral GNM (Figure 1) according to the Surface Electromyography for a Non-Invasive Assessment of Muscles (SENIAM) guidelines (28). Prior to electrode placement, the skin was cleaned with alcohol and prep gel (Nuprep, CO, USA) to minimize impedance. The raw sEMG signal was recorded at 2,000 Hz and filtered using a 4th order Butterworth band-pass filter with cutoff frequencies of 20 and 400 Hz (29, 30). The filtered sEMG data was full-wave rectified and smoothed using a moving average to estimate the sEMG linear envelope (29, 30). Furthermore, the area under the envelope was calculated to estimate the integrated EMG (iEMG) to quantify the level of muscular activity (31, 32). EMG analysis was performed using custom-made software programmed in MATLAB (The MathWorks Inc., Natick, MA, USA).

### Efficacy outcomes

**FIGURE 2:**
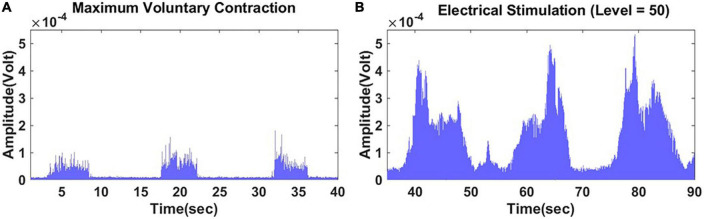
A typical case comparison between a maximum voluntary contraction and involuntary contraction of the gastrocnemius muscle assessed *via* surface electromyogram. **(A)** Three 5–10 s dorsiflexion maximum voluntary contractions. **(B)** Three 5–10 s intervals of electrical stimulation set at 50 V. Both panels having a 5–10 s relaxation period between contractions.

Lower-extremity muscle outcomes included voluntary and involuntary contraction metrics. First, in a standardized supine position (33), Ankle_s_ was determined by the average of three 5 s dorsiflexion maximum voluntary isometric contractions (MVIC) per 30 s of relaxation in-between (Figure 2) assessed with a dynamometer (RoMech Digital Hanging Scale). Second, GNM endurance [GNM_e_, defined as sustained muscle involuntary contraction (34)] in response to 5 min of E-Stim therapy was assessed with iEMG analysis.

Lower-extremity perfusion outcomes included plantar tissue oxygen saturation (SatO2), a surrogate of muscle oxygen consumption in response to E-Stim (35). SatO2 was measured using a validated Near Infra-red Spectroscopy (NIRS) camera (Snapshot NIR, KENT Imaging Inc., Calgary, AB, Canada) that detects an approximate value of real-time SatO2 level in superficial tissue. SatO2 levels were examined in the metatarsal area, including the five toes. Muscular and perfusion outcomes of the lower extremity were assessed at baseline, 3 and 9 days.

Lower-extremity functional outcome was the likelihood of falling assessment *via* Morse Fall Scale (MFS) (36). This scale is a standardized assessment performed by hospitalists at BSLMC that assesses functional aspects of the lower extremity such as ambulatory aid, gait, and transferring, among other features related to risk of falling. As the score increases, it indicates proportionally worse outcomes (low risk < 24; moderate risk 25–44; high risk > 45). The functional outcome was collected from the electronic medical records at baseline, and at the time of ICU discharge (i.e., home, hospital floor, or patient expiration) to assess overall impact of intervention on subjects. Electronic medical records were also reviewed to differentiate whether outcomes were associated with any demographic characteristics or comorbidities.

### Safety and feasibility outcomes

Safety outcomes included monitoring of vital signs (i.e., heart/respiratory rate, blood pressure, blood saturation of oxygen), and study-related adverse events (i.e., pain, skin damage, discomfort, non-compliance). Feasibility outcomes included average of patient E-Stim therapy completion, average of measured outcomes at each time point (i.e., 3 and 9 days) excluding non-study-related adverse events (i.e., death, intubation, deep vein thrombosis, rapid deterioration) (37, 38). Acceptability outcomes included interference with ongoing COVID-19 standard of care procedures (i.e., mechanical ventilation, prone rotation, PT, other clinical trials), and interaction with the ICU staff (i.e., nurses, respiratory and occupational therapists, nutritional specialists, machinery technicians).

### Sample size justification and power analysis

The sample size was estimated based on a Najafi et al. study (39), in which the effectiveness of daily lower-extremity E-Stim demonstrated a significant improvement in motor performance (Cohen effect size, *d* = 1.35). To observe the benefit of functional E-Stim (IG) to prevent or improve lower-extremity muscle outcomes compared to non-functional (CG), we conducted a power analysis following a (1) Conservative effect size (Cohen’s *d* = 0.6); (2) 80% generated power; (3) Alpha of 5%; (4) two number of groups; and (5) two repeated measurements, utilizing G *Power software (version of 3.1.6) (40). Each lower extremity was considered as an independent sample due to the variability in muscular and vascular status (41, 42).

### Statistical analysis

Shapiro–Wilk test (*p* > 0.05) was used to assess the normality of the data. Independent *t*-test was used for group comparison at baseline on normally distributed continuous demographics, clinical data, and sEMG parameters. Mann–Whitney *U* test was used if the assumption of normal distribution was not satisfied. For categorical variables, Chi-square test was used to compare between-group differences at baseline. The effect size for baseline continuous and categorical data were measured using Cohen’s *d* and Cramer’s *V*, respectively. Values ranging from 0.20 to 0.49 indicate small effects, and values between 0.50 and 0.79 indicate medium effects. Values ranging from 0.80 to 1.29 indicate large effects, and values above 1.30 indicate very large effects. Generalized estimating equations (GEE) was used to test the main effect of group (two levels: CG and IG), time [two levels: baseline, 3/9 days (muscle and perfusion outcomes), or discharge time (MFS Score)], and their interaction on the outcome measures. For all tests, an alpha level of < 0.05 was considered statistically significant. All calculations were made using IBM SPSS Statistics 27 (IBM, IL, USA).

### Ethical consideration

This study was approved by the local Institutional Review Board (IRB) at Baylor College of Medicine (Houston, TX, USA) in accordance with the Declaration of Helsinki (approval number H-47781). All participants read and signed the IRB-approved informed consent forms before initiating assessments or data collection. If the participant was cognitively impaired, consenting was performed *via* telephone call with a legal representative. The informed consent was obtained from all participants and/or their legal guardians.

## Results

### Clinical characteristics

**FIGURE 3:**
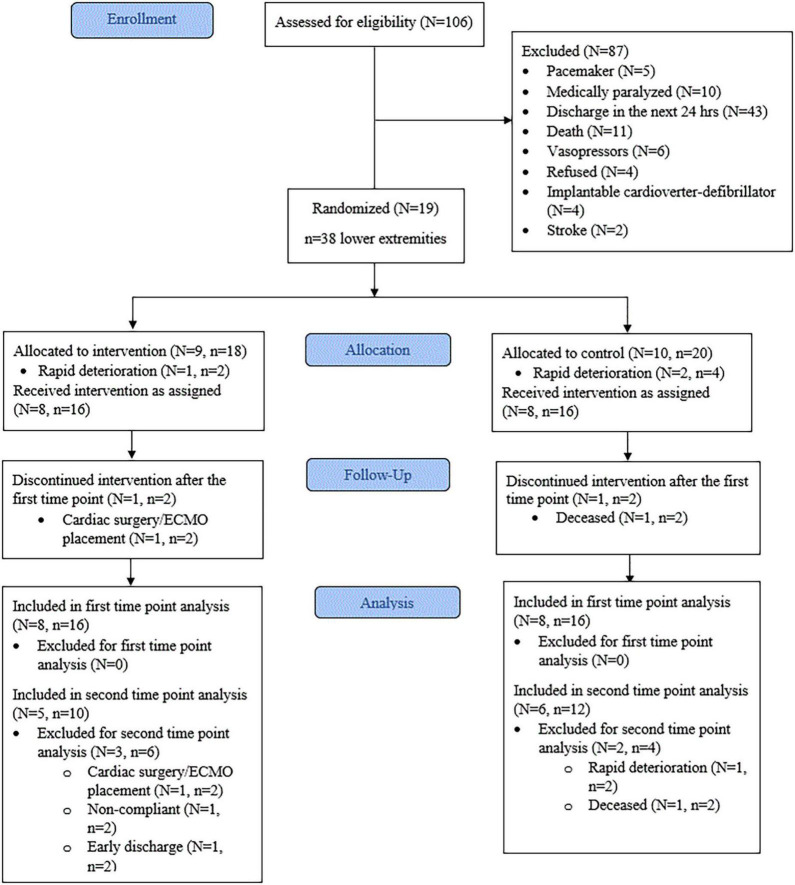
Consort flow diagram. N, number of patients. n, number of lower extremities. ECMO, extracorporeal membrane oxygenation. First time point = 3 days. Second time point = 9 days.

The progress through the phases of screening, allocation, follow-up, and data analysis is shown in Figure 3. The vast majority of patients were excluded from initial screening due to anticipated discharge from critical care within 24 h. Nineteen participants satisfied the inclusion and exclusion criteria. From these, three were withdrawn due to rapid deterioration before the mid-point (3 days), leaving a total of 16 participants (Age = 64.8 ± 9.5, *p* = 0.43, *d* = 0.40) for analysis. Therefore, eight participants (*n* = 16 lower extremities) were allocated to the CG and eight participants (*n* = 16 lower extremities) to the IG. Baseline characteristics showed the IG group had significantly higher fasting glucose (*p* < 0.01, *d* = 1.767) than the CG. All other baseline characteristics were not significantly different between the groups (Table 1). The mean time between ICU admission and delivery of E-Stim therapy was 1.8 ± 1.9 days (*p* = 0.61, *d* = 0.25). At the study enrollment, the mean body SatO2 (SpO2) was 80.53 ± 13.38% (*p* = 0.27, *d* = 0.56) in the complete cohort with all the participants (100%) undergoing corticosteroid therapy. Limited or null mobility persisted for a mean of 3.3 ± 3.5 days (*p* = 0.54, *d* = 0.38) and after 7.5 ± 5.7 days (*p* = 0.93, *d* = 0.05), 68.7% (*p* = 0.59, *V* = 0.13) of the participants began vigorous activity involvement (i.e., physical/occupational therapy, standing up, walking to chair). All others (31.3%) remained immobile during their ICU stay.

**TABLE 1:** Demographic and clinical characteristics.

	Intervention group (*N* = 8, *n* = 16)	Control group (*N* = 8, *n* = 16)	*P*-value	Effect size
**Baseline characteristics**				
Female, no.	5 (62.5)	2 (25)	0.31	2.28
Male	3 (37.5)	6 (75)		
Age, years	66.75 ± 9.81	62.88 ± 9.51	0.43	0.40
**Ethnicity, no.**				
Caucasian	1 (6.25)	1 (12.5)	0.31	0.26
African American	5 (31.3)	2 (25)		
Hispanic	9 (56.3)	5 (62.5)		
Asian	1 (6.25)	0 (0)		
BMI, kg/m^2^	28.49 ± 7.17	32.45 ± 8.04	0.31	0.52
Diabetes mellitus, no.	3 (37.5)	6 (75)	0.13	0.37
Hypertension	5 (62.5)	6 (75)	0.59	0.13
Hyperlipidemia	4 (50)	2 (25)	0.30	0.25
Acute kidney injury	1 (12.5)	1 (12.5)	1.00	0.00
Chronic kidney disease	1 (12.5)	2 (25)	0.52	0.16
Coronary artery disease	1 (12.5)	1 (12.5)	1.00	0.00
Anemia	8 (100)	6 (75)	0.13	0.37
Pneumonia	7 (87.5)	8 (100)	0.30	0.25
biPAP/CPAP use	3 (37.5)	4 (50)	0.31	0.25
Vapotherm use	3 (37.5)	5 (62.5)	0.31	0.25
Hypercoagulable state	7 (87.5)	6 (75)	0.52	0.16
Immunosuppressed status	3 (37.5)	4 (50)	0.61	0.12
Time between admission and E-Stim therapy, days	2.13 ± 1.25	1.63 ± 2.45	0.61	0.25
Total E-Stim therapy duration, days	7.00 ± 3.02	8.75 ± 3.45	0.29	0.53
Physical therapy no.	5 (62.5)	6 (75)	0.59	0.13
Deceased after study end-point, no.	4 (50%)	5 (62.5)	0.61	0.12
**Laboratory values**				
SpO2, %	78.75 ± 16.35	82.57 ± 9.82	0.27	0.56
Hb, g/Dl	11.20 ± 2.34	12.56 ± 2.37	0.26	0.57
Platelets, K/CU MM	253.00 ± 94.80	247.50 ± 146.55	0.93	0.04
WBC, K/μL	13.01 ± 3.91	9.36 ± 3.23	0.06	1.01
Glucose, mg/Dl	104.88 ± 28.08	167.38 ± 41.39	< 0.01	1.76
Creatinine, mg/Dl	1.92 ± 1.21	1.58 ± 1.23	0.58	0.27
D-dimer, MG/L FEU	4.15 ± 5.42	4.32 ± 7.11	0.96	0.02
Ferritin, ng/Ml	1360.38 ± 1345.60	2303.31 ± 1707.55	0.29	0.60
Fibrinogen, mg/Dl	528.21 ± 237.50	521.02 ± 264.60	0.95	0.02
Lactase dehydrogenase, mg/Dl	579.43 ± 138.17	907.67 ± 660.90	0.22	0.71

*N*, number of patients; *n*, number of extremities. Values are presented as mean ± standard deviation or *n* (%); BMI, body max index; biPAP, bi-level positive airway pressure; CPAP, continuous positive airway pressure; SpO2, body oxygen saturation without ventilatory assistance; Hb, hemoglobin; WBC, white blood cells. Immunosuppressed status included patients with renal or lung transplant, cancer, or autoimmune disease. The effect sizes were calculated by Cohen’s *d* (continuous) and Cramer’s *V* (categorical), respectively.

### Efficacy outcomes and longitudinal analysis

**FIGURE 4:**
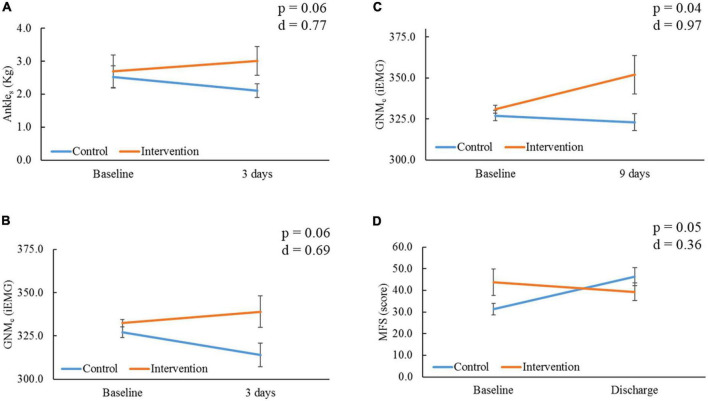
Comparison of outcomes within and between groups through time. Ankle_s_, ankle strength; kg, kilograms; GNM_e_, gastrocnemius muscle endurance; iEMG, integrated electromyography unit; MFS, Morse Fall Risk Scale. **(A)** Comparison of Ankle_s_, and **(B)** GNM_e_ within and between groups at 3 days from baseline. **(C)** Comparison of GNM_e_ within and between groups at 9 days from baseline. **(D)** Comparison of MFS within and between groups at the time of intensive care unit (ICU) discharge (18.00 ± 10.19 days) from baseline; severity is proportional to high score. *P*-value and Cohen’s *d* effect size are noted from time group interaction at each determined time point.

At 3 days, the IG showed a non-significant improvement compared to the CG with medium effect sizes for Ankle_s_ (*p* = 0.06, *d* = 0.77, Figure 4A) and GNM_e_ (*p* = 0.06, *d* = 0.69, Figure 4B), whereas the CG showed a non-significant deterioration for GNM_e_ in comparison to baseline (−3.9%, *p* = 0.08). At 9 days, the IG showed a significant improvement compared to the CG with large effect size for GNM_e_ (*p* = 0.04, *d* = 0.97, Figure 4C). In comparison to baseline, the IG’s GNM_e_ showed a significant improvement (+6.3%, *p* = 0.029). Lower-extremity oxygen consumption (SatO2) values remained stable between and within groups through time (*p* > 0.05, Table 2). At the time of ICU discharge, the IG showed a significant improvement compared to the CG with small effect size for MFS score (*p* = 0.05, *d* = 0.36, Figure 4D). In comparison to baseline, the IG’s MFS score showed a significant improvement (−12.7%, *p* = 0.05), opposite to the CG, which showed a significant worsening score (48.1%, *p* = 0.04). All other parameter comparison are shown in Table 2.

**TABLE 2:** Outcome comparison across time between both groups.

Outcomes	CG (*n* = 16)		Time effect *P*-value	Δ%	IG (*n* = 16)	Time effect *P*-value	Δ%	Time × group *P*-value (Cohen’s *d*)
**3 days**								
Ankle_s_, kg	Baseline	2.5 ± 1.2	0.15	−8	2.7 ± 1.7	0.26	42	0.06 (0.77)
	3 days	2.1 ± 0.7			3.0 ± 1.6			
GNM_e_, iEMG	Baseline	327 ± 12	0.08	−3.9	331 ± 10	0.37	1.8	0.06 (0.69)
	3 days	314 ± 27			338 ± 36			
Plantar SatO2, %	Baseline	64.9 ± 9.6	0.13	4.6	68.9 ± 6.2	0.33	2.7	0.7 (0.14)
	3 days	67.9 ± 8.0			71.3 ± 6.7			
**9 days**								
GNM_e_, iEMG	Baseline	327 ± 12	0.52	−1.5	331 ± 10	0.029	6.3	0.04 (0.97)
	9 days	323 ± 18			352 ± 37			
Plantar SatO2, %	Baseline	64.9 ± 9.6	0.92	0.4	68.9 ± 6.2	0.89	0.3	0.86 (0.06)
	9 days	67.2 ± 9.5			69.2 ± 8.9			
**Discharge**								
MFS score	Baseline	31.2 ± 7.4	0.04	48.1	43.7 ± 17	0.05	−12.7	0.05 (0.36)
	Discharge	46.2 ± 11.8			39.3 ± 11			

*N*, number of patients; *n*, number of extremities; kg, kilograms; iEMG, integrated electromyography unit. Values are presented as mean ± standard deviation Values of time effect are presented as *p*-values from generalized estimating equation models. Ankle_s_, ankle strength; GNM_e_, gastrocnemius muscle endurance; SatO2, tissue oxygen saturation; MFS, Morse Fall Risk Scale; MFS high score is proportional to severity; discharge time = 18.00 ± 10.19 days.

### Safety and feasibility outcomes

Electrical stimulation therapy did not alter vital signs nor result in adverse events during the study period. The device did not interfere with the ongoing standard of care procedures for COVID-19 ICU patients, nor cause a burden to the ICU staff. Protocol delivery showed 14/16 patients (87.5%, *n* = 28 lower extremities) were able to complete E-Stim therapy during the established study period (9 days). At the first time point (3 days), the average of measured outcomes from independent lower-extremity voluntary metrics (Ankle_s_) data excluding non-study-related adverse events (i.e., rapid deterioration = 6) was 100% (*n* = 26/26 samples), and the average for involuntary metrics (muscle endurance and SatO2) was 100% (32/32 samples). At the second time point (9 days), the average of measured outcomes from independent lower-extremity voluntary metrics (Ankle_s_) data excluding non-study-related adverse events (i.e., intubation = 10, early discharge = 2, death = 2, deep vein thrombosis = 1) was 94.1% (16/17 samples), and the average for involuntary metrics (muscle endurance and SatO2) excluding non-study-related adverse events (i.e., intubated = 4, early discharge = 2, death = 2) was 91.6% (22/24 samples). Data for MFS functional assessment at the time of ICU discharge (18.0 ± 10.2 days, *p* = 0.81, *d* = 0.11) was collected in 100% (32/32 samples) of the participants.

## Discussion

This study examined the safety and efficacy of lower-extremity E-Stim adjunctive therapy to prevent muscle deconditioning. Our main goal was to determine whether this system can improve musculoskeletal outcomes at the earliest application from ICU admission. Results suggest that patients undergoing active E-Stim to the GNM had an improvement in Ankle_s_ and muscle endurance after 3 days compared to those that utilized sham devices. Comparison at 9 days showed there was significantly higher muscle endurance in patients undergoing active stimulation compared to those that did not. We believe these findings are due to the prompt activation of muscle fibers which may deaccelerate the rapid atrophy, myofilament damage, protein synthesis, and wasting found within the first week of ICU length of stay (43). E-Stim induces non-selective recruitment and activation of both type I and type II muscle fibers which conform the GNM (44), ultimately enhancing strength and cross-sectional area (45) in immobilized patients (46). Despite wide evidence which supports the feasibility and safety of E-Stim in the ICU setting (47, 48), further exploration is needed to confirm its efficacy at improving lower-extremity musculoskeletal outcomes, particularly in patients with severe hypoxia (i.e., ICU COVID-19).

Electrical stimulation is known to excite motor units that are used for greater levels of force production (49), aiding lower-extremity muscle strength preservation for voluntary activation (50). In a recent prospective cohort study in (*n* = 5) ICU COVID-19 patients, Righetti et al. stated daily E-Stim to the quadricep muscles in mechanical ventilated patients is feasible at improving strength at 5 and 8 days per interrupted sedation assessment (21). Similar non-COVID population studies utilizing E-Stim to the peroneus longus (*n* = 24) (51) and anterior tibialis (*n* = 11) (52) found a significant improvement in ankle dorsiflexion strength. Moreover, a randomized control trial (RCT) (53) delivering E-Stim to the GNM of ICU patients (*n* = 36) showed an improvement in strength at 9 days. The present RCT in ICU COVID-19 patients undergoing active E-Stim to the GNM showed an improvement in ankle dorsiflexion strength compared to controls with a medium effect size (*p* = 0.06, *d* = 0.77, Figure 4A) after 3 days of starting therapy. Unfortunately, results at 9 days were limited due to the high morbimortality status (i.e., deep vein thrombosis, intubation, death) impeding patients from performing voluntary tests, and limiting further assessment post-sedation.

2019 Novel Coronavirus reviews have also claimed E-Stim therapy may improve muscle endurance (18); however, evidence is supported by different types of immobilized populations (54). Hence, Veldman et al. suggested that E-Stim may result in a fast-to-slow muscle fiber type transition, which could potentially enhance endurance in patients with severe weakness unable to perform voluntary contractions (i.e., cardiorespiratory, critically illness) (55). However, due to the challenging objective assessment of muscle endurance in patients with severe illness, these outcomes were functionally assessed (i.e., waking distance movement) after recovery. In our study, we assessed real-time muscle endurance in the ICU setting utilizing electromyography (56). This test is optimal for assessment of muscle endurance and weakness as it offers a way to study the myoelectric features of neuromuscular activation associated with E-Stim (57). That said, the present study showed the IG had a higher improvement in GNM_e_ with medium effect size at 3 days (*p* = 0.06, *d* = 0.69, Figure 4B) than the CG, yet a significant improvement with a large effect size at 9 days (*p* = 0.04, *d* = 0.97, Figure 4C) by increasing 6.3% (*p* = 0.029) from baseline (Table 2). This was especially noteworthy since all patients had null mobility over the first ∼3 days from ICU admission, avoiding any confounding effect of physical or nutritional therapy. This suggest that daily E-Stim may gradually improve GNM_e_ in ICU COVID-19 patients.

Multiple studies (58) have suggested applying E-Stim therapy immediately after ICU admission to prevent lower-extremity neuromuscular damage in ICU patients (59–61). The physiology behind this suggestion relies on the fact that early introduction to E-Stim can ensure early activation/contraction of the motor unit (15). This is important because there is an abrupt decline in amplitude of nerve action potential and motor depolarization within 24 h from ICU admission (62). In ICU COVID-19 patients, there is an additional degenerative transformation and shrinkage of skeletal muscle due to sarcopenia, oxidative stress, and hyper-catabolism induced by cytokine storms and malnutrition (11, 63). In the present study, E-Stim was provided within ∼1.8 days from ICU admission; thus, an early involuntary contraction of motor units may have led the IG muscle outcomes to improve as early as 3 days. However, we believe therapy should be provided for prolonged periods, even after ICU discharge. Further studies are needed to explore musculoskeletal outcomes with the continuous use of E-Stim after hospital discharge.

Another consequence of immobile status, loss of voluntary contraction, and weakness acquired from the ICU is physical function impairment (64–66). A recent systematic review about post-ICU syndrome (67) reported significant functional disability due to lower-extremity problems during the first year following critical illness. The present study utilized the MFS (36) that evaluates ambulatory aid, gait, and transferring, among other functional aspects. Despite some patients losing consciousness during the study period (*N* = 6) and high-risk morbimortality, E-Stim was safely and continuously delivered in 87.5% (*n* = 28 lower extremities) of the cohort for a 9 days period without interfering with the responsibilities of the hospital staff. The short-time therapy effect was reflected in the significantly lower likelihood of falling in the IG compared to the CG (*p* = 0.05, Figure 4D) at the time of ICU discharge. Nonetheless, a longer follow-up period with larger sample sizes targeting functional objective measurements is warranted to assess limb functionality in critically ill COVID-19 patients undergoing E-Stim therapy.

Under E-Stim therapy, oxygen consumption of the lower extremity increases to supply energy to the lower-extremity muscles and thus maintain isometric muscle contraction (35, 68). At hypoxic levels, glycogen substitutes oxygen for energy supplementation *via* the anaerobic metabolism pathway (69) that, when depleted, may result in muscle fatigue and subsequent injury (70). Although no study has explored the effect of E-Stim on the tissue perfusion in the lower extremities of ICU COVID-19 patients (71), Gerovasili et al. examined it the thenar muscle of (*n* = 29) ICU patients. With a provoked vascular occlusion, they found that mean SatO2 assessed with Near Infra-red Spectroscopy (NIRS) did not differ before and after E-Stim therapy. With the severe hypoxia and blood oxyhemoglobin disassociation that critically ill COVID-19 patients present (72), one could expect this population may be more susceptible to muscle perfusion deterioration after undergoing stress (73). In the present RCT, the IG’s lower-extremity distal perfusion showed a similar pattern to the CG by remaining stable during the study period, meaning that daily E-Stim did not alter the level of muscle oxygen consumption in patients with severe hypoxia. However, studies evaluating SatO2 before and after 1 h E-Stim are needed to explore the real-time effects of COVID-19 in lower-extremity muscle perfusion.

We acknowledge the main limitation of this study is the small sample size, which may be underpowered for some of the outcomes. However, from the feasibility standpoint, we successfully collected all outcomes (i.e., Ankle_s_, muscle endurance, and muscle perfusion), excluding those who underwent non-study-related adverse events, in 100% of the available samples at the 3 days time point, and 91.6% at the 9 days time point. Based on the observed effect sizes (*d* = 0.69–0.77, Table 2) at the 3 days time point, the available 26 samples (lower extremities) resulted in a generated power in range of 92–96% for Ankle_s_, whereas for muscle endurance, the available 32 samples (lower extremities) resulted in a generated power in range of 97–99%. At the 9 days time point, the generated power for muscle endurance was greater than 80% (available samples = 22, *d* = 0.97, Table 2). However, the power was insufficient for Ankle_s_ (less than 80%) because of the reduced available samples (*n* = 15) in patients with deep vein thrombosis, intubation, or death due to COVID-19; thus, were not reported. This study was preventative; therefore, patient selection focused on those at high risk of muscle deconditioning but not clinically diagnosed with established guidelines for myopathy or neuropathy. COVID-19 variants were not reported. Creatinine phosphokinase, serum lactate, nor blood indicators for muscle damage were measured. SatO2 was not directly measured from the GNM. There were no other muscles stimulated or assessed. The duration of follow-up was short due to the high mortality rate in this particular population. Despite these limitations, the observed medium effects for benefit of E-Stim, ease of administration without overwhelming the nursing staff, and high acceptability encourage future studies to confirm the observed effects in preventing muscle deconditioning among clinically ill patients who require prolonged bed rest.

## Conclusion

Our study supports the safety and efficacy of E-Stim in the ICU setting to prevent deterioration of lower-extremity muscle weakness in critically ill COVID-19 patients. This adjunctive therapy may provide a potential benefit for gastrocnemius muscle endurance and Ankle_s_, thus, possibly aid on the prevention of functional sequelae in critically ill bedbound patients or those with similar characteristics of severe hypoxia or low SpO2. This is also true given the fact the benefit was observed in those intubated patients who continued to receive E-Stim therapy during their hospital length of stay. The benefits of E-Stim rely on the rapid involvement of therapy at the time of ICU admission. However, E-Stim does not replace PT, but rather enhances gastrocnemius muscle endurance and Ankle_s_ as an adjunctive treatment. Moreover, a portable and practical system that is easy to use does not interfere with the daily duties of the ICU staff. In addition, E-Stim did not alter vital signs or lower-extremity oxygen consumption, nor did it show adverse events during the study period. Further studies with larger sample sizes and longer follow-ups are warranted to examine the effectiveness of E-Stim to prevent muscle deconditioning in critically ill COVID-19 patients.

## Data availability statement

The data that support the findings of this study are not publicly available but are available from the corresponding author BN, najafi.bijan@gmail.com upon reasonable request.

## Ethics statement

The studies involving human participants were reviewed and approved by the Institutional Review Board for Human Subject Research for Baylor College of Medicine and Affiliated Hospitals (BCM IRB). The patients/participants provided their written informed consent to participate in this study. If the participant was cognitively impaired, consenting was performed via telephone call with a legal representative. The informed consent was obtained from all participants and/or their legal guardians.

## Author contributions

BN, AZ-R, NR, MS, and JPH: concept and study design. AZ-R, NR, RM, RB, ML, and AB: acquisition, analysis, and interpretation of the data. AZ-R, RM, RB, ML, and AB: drafting the manuscript. BN, MS, and JPH: critical revision of the manuscript for important intellectual context. RM: statistical analysis. BN: obtained the funding. AZ-R, NR, and RB: administrative, technical, or material support. BN, MS, and JPH: supervision. All authors contributed to the article and approved the submitted version.
